# Cytogenetic Analysis, Heterochromatin Characterization and Location of the rDNA Genes of *Hycleus scutellatus* (Coleoptera, Meloidae); A Species with an Unexpected High Number of rDNA Clusters

**DOI:** 10.3390/insects12050385

**Published:** 2021-04-26

**Authors:** Laura Ruiz-Torres, Pablo Mora, Areli Ruiz-Mena, Jesús Vela, Francisco J. Mancebo, Eugenia E. Montiel, Teresa Palomeque, Pedro Lorite

**Affiliations:** Department of Experimental Biology, Genetics Area, University of Jaén, 23071 Jaén, Spain; lrtorres@ujaen.es (L.R.-T.); pmora@ujaen.es (P.M.); armena@ujaen.es (A.R.-M.); jvela@ujaen.es (J.V.); fj.mancebo@isciii.es (F.J.M.); emontiel@ujaen.es (E.E.M.); tpalome@ujaen.es (T.P.)

**Keywords:** Coleoptera, Meloidae, blister beetles, cytogenetics, nucleolar organizer regions, rDNA clusters, satellite DNA, heterochromatin, in situ hybridization

## Abstract

**Simple Summary:**

The family Meloidae contains approximately 3000 species, commonly known as blister beetles for their ability to secrete a substance called cantharidin, which causes irritation and blistering in contact with animal or human skin. In recent years there have been numerous studies focused on the anticancer action of cantharidin and its derivatives. Despite the recent interest in blister beetles, cytogenetic and molecular studies in this group are scarce and most of them use only classical chromosome staining techniques. The main aim of our study was to provide new information in Meloidae. In this study, cytogenetic and molecular analyses were applied for the first time in the family Meloidae. We applied fluorescence staining with DAPI and the position of ribosomal DNA in *Hycleus scutellatus* was mapped by FISH. *Hycleus* is one of the most species-rich genera of Meloidae but no cytogenetic data have yet been published for this particular genus. Additionally, we isolated a satellite DNA family located within the pericentromeric regions of all chromosomes. The results obtained in this study may be a suitable starting point to initiate more extensive cytogenetic analyses in this important species-rich genus, and in the family Meloidae in general.

**Abstract:**

Meloidae are commonly known as blister beetles, so called for the secretion of cantharidin, a toxic substance that causes irritation and blistering. There has been a recent increase in the interest of the cantharidin anticancer potential of this insect group. Cytogenetic and molecular data in this group are scarce. In this study, we performed a karyotype analysis of *Hycleus scutellatus*, an endemic species of the Iberian Peninsula. We determined its chromosome number, 2n = 20, as well as the presence of the X and Y sex chromosomes. In addition to a karyotype analysis, we carried out DAPI staining. By fluorescence in situ hybridization we mapped the rDNA clusters on 12 different chromosomes. Compared to others, this species shows an unusually high number of chromosomes carrying rDNA. This is one of the highest numbers of rDNA sites found in the Polyphaga suborder (Coleoptera). Additionally, we isolated a satellite DNA family (Hyscu-H), which was located within the pericentromeric regions of all chromosomes, including the sex chromosomes. The results suggest that Hyscu-H is likely to be one of the most abundant satellite DNA repeats in *H. scutellatus*.

## 1. Introduction

The family Meloidae, commonly known as blister beetles or the Spanish fly, contains approximately 3000 species, divided among 125 genera and four subfamilies [1]. The name “blister beetles” refers to the ability to secrete a body fluid called cantharidin, a chemical that is able to cause irritation and blistering when it contacts animal skin. Males synthesize cantharidin as a defensive mechanism and it is also given to the females as a copulatory gift during mating [2].

Crushed beetles of the species *Lytta vesicatoria* and other Meloidae species were used since ancient times as an aphrodisiac. Cantharidin causes vascular congestion and inflammation of the male genitourinary tract. However, numerous studies advice against its use since cantharidin is a potent poison that generates numerous toxic effects [3,4]. Currently, cantharidin is only used for medical purposes as a topical solution for warts and molluscum [4]. Blister beetles have been used in traditional Chinese medicine for more than 2000 years, and nowadays are still used as a folk medicine [5]. In recent years there have been numerous studies focused on the anticancer action of cantharidin and its derivatives [6,7]. The recent interest in cantharidin and in its biosynthetic pathways has led to the genome sequencing of two of the most important blister beetles in traditional Chinese medicine; *Hycleus cichorii* and *Hycleus phaleratus* [8]. Such genome assemblies could help to understand the biological synthesis pathways and evolution of cantharidin in blister beetles.

Despite the recent interest in blister beetles, cytogenetic studies in this group are scarce. Currently, there are data on less than 30 species [9,10]. Most of the studies were conducted before and during the 1960s and 1970s. Only conventional staining methods were used in these studies, and they revealed only the chromosome number and the sex chromosome system (Table 1). A more recent study showed that the insect telomeric repeat, (TTAGG)*n*, was substituted for (TCAGG)*n* in all tested Tenebrionoidea species, among them a non-classified blister beetle from the genus *Mylabris* [11]. C-banding and silver staining of the nucleolar organizer regions (NORs) have been carried out only in *Epicauta atomaria* [12]. C-banding in this species demonstrated the presence of constitutive heterochromatin within the pericentromeric regions of the chromosomes, but also in subtelomeric and interstitial positions. The main component of constitutive heterochromatin is satellite DNA (satDNA), a highly repetitive, non-coding DNA. Satellite DNA usually consists of a single sequence (repeat unit or monomer) arranged in a head-to-tail manner many times across the genome. The monomer lengths of satDNA repeats are highly variable, ranging from several bp for simple repeats, to several kb for complex repeats [13,14]. The role of satDNA has been debated for a long time. However, nowadays there are multiple lines of evidence demonstrating the importance of satDNA in centromere function, the formation of heterochromatin and chromosome pairing [15,16]. It is quite usual that species or other taxa share satDNA families [13]. SatDNA generally shows a pattern of concerted evolution, with higher intraspecific than interspecific similarity [17]. Although the use of satDNA as a phylogenetic marker is controversial, there are numerous examples in which the evolution of satDNA reflects species relationships in phylogenetic analyses [18,19]. Likewise, the molecular cytogenetic analysis of the nucleolar organizer regions (NORs) has been widely used in the study of chromosomal evolution and phylogeny among related species [20,21]. Traditionally, the chromosomal localization of the rDNA clusters has been performed using silver staining techniques. However, this technique shows two drawbacks: it only stains active NORs, that is, those that were transcribed during the previous interface [22], and in some cases stains heterochromatic regions [23,24]. On the contrary, fluorescent in situ hybridization (FISH) techniques allow access to the genes themselves, regardless of their state of expression, even allowing its detection and study in interphase nuclei [25]. In fact, it is widely accepted that FISH is a uniquely effective tool for physically mapping rDNA genes [26,27].

*Hycleus* is one of the most speciose genera of blister beetles, including 500 species that are mainly distributed throughout the Old World [52]. In spite of this pattern, there are currently no cytogenetic data for any species of this genus. Thus, this study, which focuses on *H. scutellatus* Rosenhauer, 1856, an endemic species of the Iberian Peninsula [53], represents the first cytological examination of any species within this group. In addition to karyotype analysis, we conducted DAPI staining and FISH to visualize the positions of the rDNA clusters. We also identified a satDNA repeat that was located within the pericentromeric regions of all chromosomes, making it probably one of the most abundant satDNAs in this insect. Moreover, this study represents the first of its kind for any species belonging to Meloidae. Our results represent a suitable starting point for more extensive cytogenetic analyses in this important species-rich genus.

## 2. Materials and Methods

### 2.1. Chromosome Preparations and DAPI Staining

The specimens of *H. scutellatus* were collected in Alcudia de Guadix, Spain (37.79 N, −3.78 W). *H. scutellatus* is not an endangered or protected species and no specific permission was required for its collection. Testes were dissected from males for chromosome preparation. After dissection, the bodies were preserved in 100% ethanol at −20 °C and used subsequently for genomic DNA extraction. Chromosomes slides were prepared as described in Lorite et al. [54]. Chromosome spreads were stained with Giemsa or 4′-6-diamino-2-fenil-indol (DAPI) [55], and they were analyzed with an Olympus (Hamburg, Germany) BX51 fluorescence microscope equipped with an Olympus DP70 camera. Images were processed using Adobe Photoshop CS4 (Adobe Systems, San Jose, CA, USA).

### 2.2. Extraction of Genomic DNA, Isolation of Repetitive DNA and Computer Analysis

Total genomic DNA was isolated using the NucleoSpin Tissue kit (Machery-Nagel GmbH & Co., Düren, Germany) following instructions provided by the company. For repetitive DNA isolation, DNA was digested overnight with a battery of restriction endonucleases using 4 U/μg DNA. Digested DNA was separated in 2% agarose gels. Fragments of approximately 350 and 700 bp, generated by digestion with *Hpa*I (GTT/AAC), were eluted from the agarose gel and ligated into the pUC19 vector that was linearized with the blunt end-producing endonuclease *Sma*I (CCC/GGG). Ligation reactions were used to transform competent *Escherichia coli* DH5α bacteria (Zymo Research, Orange, CA, USA). Recombinant cells were selected from colonies grown on LB/ampicillin/IPTG/X-Gal plates. A portion of the eluted fragments were labeled using a DIG DNA labeling kit (Roche, Basel, Switzerland) and used for plasmid screening. Recombinant plasmids yielding positive hybridization signals were sequenced on both strands using the universal primers SP6 and T7. The identified satDNA family was called Hyscu-H. Multiple-sequence alignments of the identified repeats were performed using the CLUSTALW software. Distance analysis was conducted with MEGA software version X [56]. The sequence data were analyzed and compared with the DNA databases using NCBI’s BLAST.

The satDNA sequences were analyzed using a predictive model of sequence-dependent DNA bending. The magnitude of DNA curvature was calculated with the BEND server algorithm of Goodsell and Dickerson [57] using the bend.it server (http://pongor.itk.ppke.hu/dna/bend_it.html#/bendit_intro, (accessed on 24 April 2021) [58]). The curvature values are presented as the deflection angle per 10.5 residue helical turn (1°/bp = 10.5/helical turn).

### 2.3. Dot-Blot Hybridization and Fluorescence In Situ Hybridization.

Dot-blot hybridization was used to estimate the amount of the Hyscu-H satDNA in the *H. scutellatus* genome [59,60]. Inserts of the recombinant Hyscu-H-20 and Hyscu-H-21 plasmids were labeled with DIG as indicated above and used as a probe in the dot-blot hybridization.

Physical location of the rDNA clusters was determined by DNA FISH. The plasmid pDmra.51#1, with a noninterrupted 11.5 kb rDNA unit containing 18S and 28S genes of *Drosophila melanogaster* [61], was used as a probe. For location of the Hyscu-H satDNA family repeats, the inserts of the recombinant plasmids were used as a probe. Probes were labeled with biotin-16-dUTP using the biotin nick translation kit (Roche Diagnostics GmbH, Mannheim, Germany). FISH was carried out following the procedure described by Palomeque et al. [62] using the biotin-labeled probe (2 ng probe/mL, 50% formamide). FISH probe detection was performed using the avidin-FITC/anti-avidin-biotin system with two amplification rounds for the satDNA probe and three rounds for the rDNA probe. Slides were mounted using VECTASHIELD^®^ with DAPI (Vector Labs, Burlingame, CA, USA).

## 3. Results and Discussion

**Figure 1:**
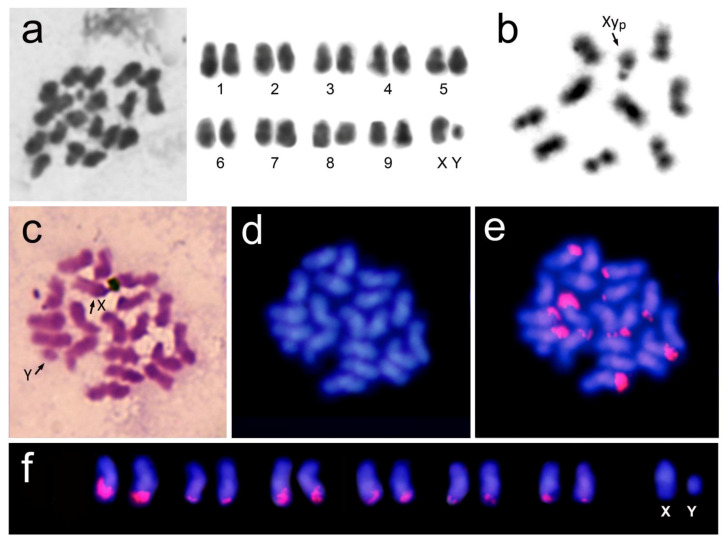
(**a**) Giemsa staining of mitotic chromosomes in the male karyotype of *H. scutellatus*; (**b**) Giemsa staining of meiotic chromosomes at metaphase I showing the Xyp parachute system; and (**c**) male mitotic chromosomes after Giemsa staining. (**d**) The same metaphase after DAPI staining and after (**e**) FISH using rDNA as a probe, showing positive hybridization signals (in pink) on six pairs of chromosomes. (**f**) Selected pairs of autosomes carrying the rDNA. Sex chromosomes are also shown. The X and Y chromosomes are indicated by arrows.

We determined that the diploid chromosome number of *Hycleus scutellatus* was 2n = 20, with nine pair of autosomes and the sex chromosomes, X and Y. All autosomes and the X chromosome were meta- or submetacentric; in contrast the Y chromosome was minute and thus its morphology could not be determined (Figure 1a,c). Cytogenetic studies in the Meloidae family are scarce and currently the karyotypes of only nineteen species are known (Table 1). In general, Meloidae species have a chromosome number of 2n = 20, which is also the most frequent in the suborder Polyphaga, although six exceptions have been described in the subfamily Meloinae, with chromosome numbers of 2n = 22 and 2n = 24 (Table 1). 

In meiosis I, the sex chromosomes are associated with one another, showing the typical Xyp parachute configuration (Figure 1b). This reflects a non-chiasmata association between a large, biarmed X chromosome and a minute Y chromosome (written in lowercase “y”) [35,51]. The meioformula 9 + Xyp found in *H. scutellatus* is typical for most of the Meloidae species (Table 1). Although the Xyp configuration is the most common and ancient sex chromosome system within the Coleoptera order [35], X0 and other sex chromosome systems have also been found in this group [63]. Likewise, other sex chromosome configurations, including XY and Xyr, are also present in Meloidae. In the Xyr system the large X chromosome and the small Y chromosome show a rod-shaped “end-to-end” pairing [35,51]. However, Xyp and Xyr systems cannot be reliably discriminated and some reported Xyr cases probably are Xyp [64]. In fact, the two systems have been reported for the same species, as in *Mylabris pustulata* or in *Mylabris thunbergi* (Table 1). In *Epicauta atomaria*, a sex chromosome polymorphism for the presence of one or several Y chromosomes has been described, with males having Xyp, Xyyp or Xyyyp [31]. 

The DAPI fluorochrome binds preferentially to A + T-rich DNA. Satellite DNA in beetles is generally A + T-rich. Therefore, DAPI staining often reveals the same regions that were stained by C-bands [59,65,66,67]. After DAPI staining, the mitotic chromosomes of *H. scutellatus* showed a strong signal within the pericentromeric regions of all of the chromosomes, including the X and Y sex chromosomes (Figure 1d), showing that the pericentromeric heterochromatin in this species is A + T-rich. On the other hand, there are other beetle species in which DAPI staining produces homogeneous staining of the chromosomes [68,69]. 

According to the published literature, there is no well-defined NOR (nucleolar organizing region) distribution pattern for any of the families of the Coleoptera order. This order includes approximately 387,000 species [70]. Of these, only in 372 species has the rDNA gene distribution pattern been studied, by using silver staining or FISH [21]. Schneider et al. [63] reviewed this pattern in 190 coleopteran species. According to the authors, most examined species showed two clusters of rDNA located on a pair of autosomal chromosomes (82 and 63% in Adephaga and Polyphaga suborders, respectively). However, there are numerous exceptions. For example, in several species of the genera *Megacephala* and *Zabrus*, a variable and high number of chromosomes carrying an rDNA cluster were found (1–8 and 2–12, respectively). Both of these genera (Adephaga suborder), include species with very different chromosomal numbers (2n = 12–29 and 2n = 57–63, respectively) and both include species with X0 as the sex chromosome system [63]. In the Polyphaga suborder, the distribution of rDNA sites has been studied especially in the family Scarabaeidae [20,63,71,72]. Most of the species in this family show two NORs located on an autosomal pair, although there is wide variability. For example, *Bubas bison* has eight chromosomes with rDNA genes [24]. Similarly, *Coprophanaeus ensifer* shows rDNA sites on seven autosomal bivalents and on the X chromosome [71]. Dutrillaux and Dutrillaux [72], in a large study with 82 species, reported that NORs were frequently located on two acrocentric autosomes, although their presence on the X chromosome was also quite common. Lopes et al. [21] analyzed 11 species of the Cassidinae subfamily (Chrysomelidae), reporting that most of the species also had rDNA sites on one pair of autosomes. Hitherto, there is only one study of NORs in the Meloidae family, carried out in *Epicauta atomaria*. In this species, silver staining of pachytene nuclei showed that the nucleolar material was associated with the 7th and Xy bivalents [12]. However, the authors indicated that silver nitrate impregnation may not show NORs location since the nucleolar material could be transferred to these bivalents during the early prophase and that the presence of NORs could only be confirmed by in situ hybridization using rDNA as a probe. FISH in *Hycleus scutellatus* shows the existence of 12 rDNA sites located at the terminal region of the long arm of six pairs of autosomes (Figure 1e,f). This is one of the highest numbers of NORs found in any member of the Polyphaga suborder. There are several hypotheses to explain this high number of rDNA clusters. This fact has been related to evolutionary changes such as Robertsonian translocations and especially to the type of technique used in the detection of NORs. It is quite accepted that silver staining only highlights active NORs, while FISH detects both active and inactive rDNA sequences. Consequently, estimates based on the latter method would be higher [20,71,72]. In fact, several Coleoptera species showed different results when their NORs were studied using silver impregnation or FISH [69,72,73,74]. Hirai [75] recently analyzed the different chromosomal observations using FISH and silver staining techniques obtained so far in organisms as different as primates and Australian bulldog ants. In ants, the increase in chromosomal number by centric fission is a frequent evolutionary mechanism. The author suggested that the transcriptional activity and genomic spread of rDNA clusters varies between organisms and depends on genomic structures [75,76]. Regardless of the above information, *Hycleus scutellatus* and *Coprophanaeus ensifer* have so far the highest number of rDNA sites in Coleoptera. In addition, both species have a low chromosome number, 2n = 20, which is very common in Coleoptera. Sproul et al. [25], in other coleopteran species and using genome sequencing data and FISH, analyzed the high variation in the number of rDNA genes and in the rDNA sites and its implication in the evolution of related species. It will be important to analyze more species of the family Meloidae in order to know whether a high number of rDNA sites is a synapomorphic trait that is characteristic of species belonging to this family, as in other coleopteran species, or if this characteristic is unique to *H. scutellatus.*

**Figure 2:**
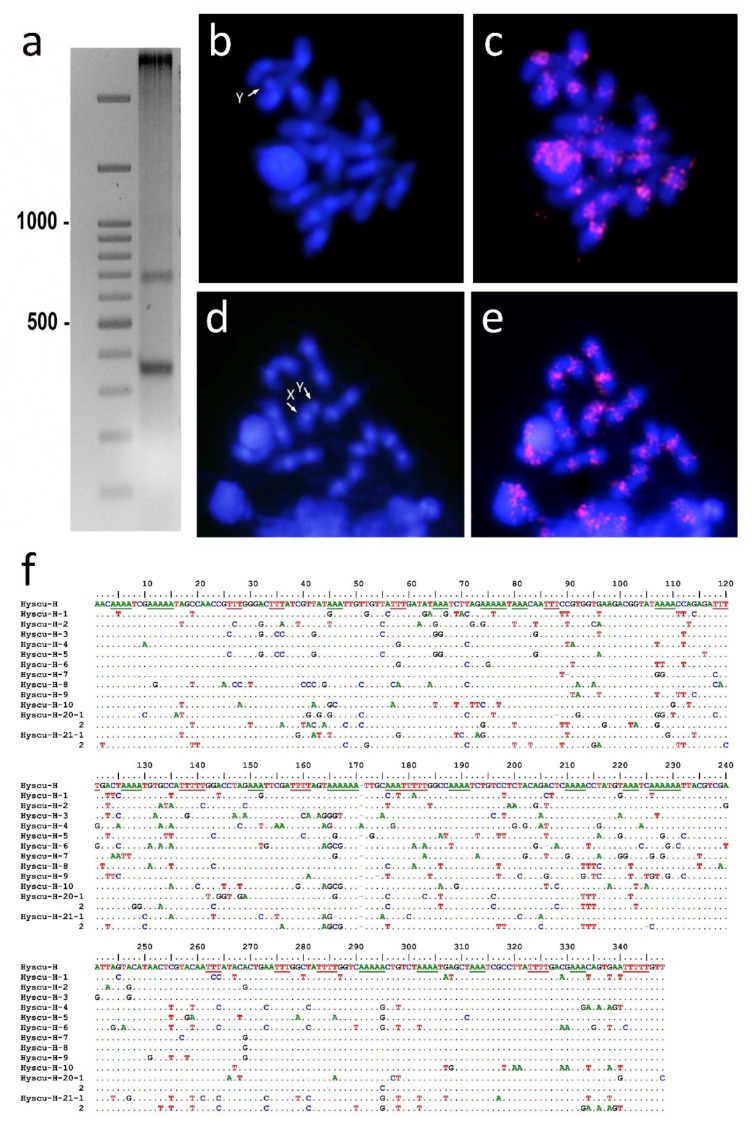
(**a**) Electrophoresis of *H. scutellatus* gDNA in a 2% agarose gel after digestion with *Hpa*I, revealing a band of repetitive DNA of approximately 350 bp and another of approximately 700 bp. The numbers on the left indicate the size in bp of the marker DNA. (**b**) Male mitotic chromosomes stained with DAPI and (**c**) subsequently FISH with the Hyscu-H satDNA probe. (**d**) Male meiotic chromosomes stained with DAPI and (**e**) subsequently FISH with the Hyscu-H satDNA probe. (**f**) Multiple sequence alignment of all sequenced monomers from the Hyscu-H satDNA family and the consensus sequence derived from them. The sequences designated 1 and 2 are monomeric units from the same dimer clone. Underlined nucleotides highlight periodic A or T runs of three or more nucleotides. The X and Y chromosomes are indicated by arrows.

Genomic DNA from *H. scutellatus* was cleaved with several restriction enzymes and separated on a 2% agarose gel. DNA digestion with *Hpa*I (GTT/AAC) generated a typical ladder pattern with a monomer length of approximately 350 bp (Figure 2a). Twelve recombinant plasmids, including ten monomers and two dimers, were sequenced. The consensus sequence of these clones was 347 bp in length. We named this family of satDNA Hyscu-H (Hy = *Hycleus*, scu = *scutelatus*, H = *Hpa*I) (GenBank accession numbers MW711195 to MW711206). The alignment of the cloned Hyscu-H repeats is shown in Figure 2f. The similarity among these repeats ranges between 79 and 88%. The observed variation among these repeats is mainly due to indels and single nucleotide substitutions. Some of the observed mutations are present in only one of the cloned repeats but some of them are shared among several repeats. These mutations could have been generated by independent events, but more probably are due to single mutations that have been extended from one sequence to another. This process can lead to homogenization of the different monomers [77]. Dot-blot hybridization showed that the Hyscu-H satDNA family comprises approximately 15% of the genome (data not shown). As mentioned above, the genomes of two *Hycleus* species (*H. cichorii* and *H. phaleratus*) have been recently sequenced at scaffold level [8]. Searches in the SRA files of these genomes did not reveal reads with similarity to Hyscu-H satDNA. Searches in other nucleotide sequence databases also failed to find any substantial similarity to the Hyscu-H satDNA. The results indicate that Hyscu-H is likely to be species-specific or that it is not shared in all species of the genus *Hycleus*.

**Figure 3:**
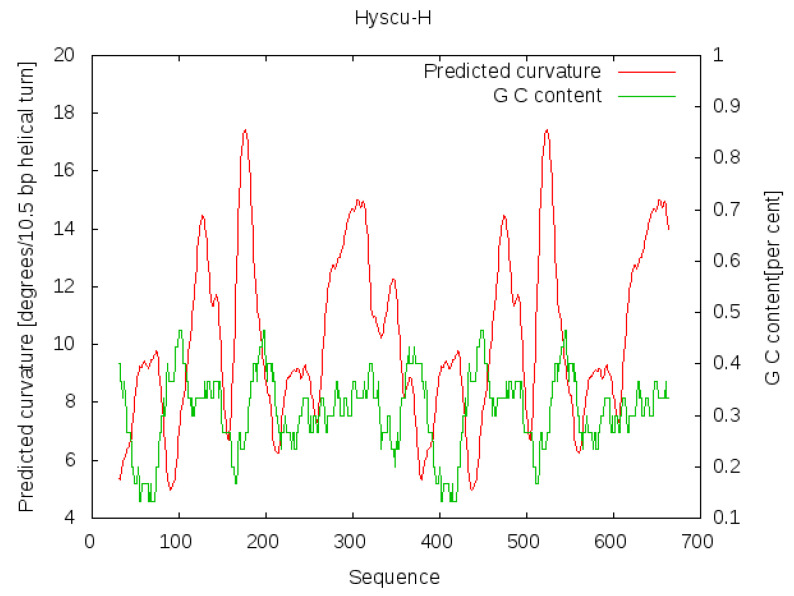
The curvature propensity and G + C content of a dimeric repeat of the Hyscu-H satDNA family.

The A + T richness of the Hyscu-H consensus sequence is 70%. The A and T residues are not randomly distributed along the consensus sequence; half of them are part of the A or T runs of three or more nucleotides. The A + T richness is a common characteristic of the satDNA in other Coleoptera and other insect groups [13]. A + T richness and the presence of periodic A or T runs tend to correspond with satDNA curvature [78,79,80,81]. Satellite DNAs with points of local curvature show anomalous electrophoretic mobility in polyacrylamide gels, with electrophoretic migration being slower than expected based on sequence length [79,82,83]. The satDNA curvature, or its potential bendability, is thought to be related to heterochromatin organization, it specifically being necessary for the packing of DNA into heterochromatin [80,84,85]. The analysis of bendability/curvature propensity was applied to a Hyscu-H dimer in order to cover the whole satellite length. Six high potential curvature peaks were observed (Figure 3); four of them had curvature propensities over 12 (around positions 130, 180, 320 and 355) and two had curvature propensities of approximately 9–9.5 (around positions 70 and 200). These magnitudes of curvature have been experimentally associated with a retarded DNA electrophoretic mobility in other insect satDNAs [81,86].

FISH using Hyscu-H as a probe showed positive hybridization signals in all chromosomes including the sex chromosomes. The location of these hybridization signals coincides with the DAPI-positive staining regions located on the pericentromeric regions (Figure 2b,c,e,f). The amount of Hyscu-H satDNA in the *H. scutellatus* genome indicates that this satDNA is likely to be one of the more abundant satDNAs in this species. However, non-uniform heterochromatin staining after FISH suggests that there are likely to be other types of repetitive DNAs in the pericentromeric regions, as is the case in other insect and non-insect eukaryotic species [87,88,89]. The characterization of new repetitive DNAs by using tools based on cytogenomics (Next Generation Sequencing in combination with FISH) will facilitate descriptions of heterochromatin composition in *H. scutellatus*.

## Figures and Tables

**Table 1:** Known chromosome numbers and sex chromosome system in species belonging to the Meloidae family.

Species	Meioformula	References
**Subfamily Meloinae**		
*Cyaneolytta n*. sp.	2n = 20, 9 + Xyp	[28,29]
*Epicauta anthracina* Erichson, 1848	2n = 20, 9 + Xyp	[30]
*Epicauta atomaria* Germar, 1821	2n = 20, 9 + Xyp	[12,31,32]
	2n = 21, 9 + Xyyp	[31]
	2n = 22, 9 + Xyyyp	[31]
*Epicauta cinerea* Forster, 1771	2n = 20, 9 + XYp	[33]
*Epicauta grammica* Fischer, 1827	2n = 24, 11 + Xyp	[34]
*Epicauta isthmica* Werner, 1949	2n = 20, 9 + Xyp	[34]
*Epicauta murina* LeConte, 1853	2n = 20, 9 + Xyp	[35]
*Epicauta n*. sp.	2n = 20, 9 + Xyp	[34]
*Epicauta pennsylvanica* Borchmann, 1917	2n = 20, 9 + XYp	[33]
*Epicauta picta* Laporte de Castelnau, 1840	2n = 20, 9 + Xyp	[36,37] (as *Lytta picta*)
*Epicauta pluvialis* Borchmann, 1930	2n = 20, 9 + Xyp	[31]
*Epicauta rosilloi* Martinez, 1952	2n = 20, 9 + Xyp	[31]
*Epicauta rufipedes* Dugés, 1870	2n = 20, 9 + Xyp	[34]
*Hycleus scutellatus* Rosenhauer, 1856	2n = 20, 9 + Xyp	Current study
*Meloe* sp.	2n = 20, 9 + Xyp	[38]
*Mylabris balteata* Pallas 1782	2n = 20, 9 + Xyr	[36,37]
*Mylabris himalayaensis* Saha, 1979	2n = 22, 10 + Xyp	[39,40] (as *M. himalayica*)
*Mylabris macilenta* Marseul, 1873	2n = 22, 10 + Xyp	[41]
*Mylabris phalerata* Pallas, 1781	2n = 22, 10 + Xyp	[36,42,43,44,45,46] (as *M. phalerta* or *M. phalcrata*)
*Mylabris pustulata* Thunberg, 1821	2n = 22, 10 + Xyp	[10,38,40,44,46,47,48]
*Mylabris thunbergi* Billberg, 1813	2n = 22, 10 + Xyp	[48,49,50]
*Paniculolytta sanguineoguttata* Haag-Rutenberg, 1880	2n = 20, 9 + Xyp	[34]
*Pyrota decorata* Haag-Rutenberg, 1880	2n = 20, 9 + Xyp	[34]
*Psalydolytta* sp.nr.*rouxi*	2n = 20, 9 + Xyp	[28,29]
*Sybaris praeustus* Redtenbacher, 1844	2n = 20, 9 + Xyp	[41] (as *S. paraeustus*)
*Sybaris testaceus* Fabricius, 1792	2n = 20, 9 + Xyp	[36,37]
**Subfamily Tetraonycinae**		
*Tetraonyx frontalis* Chevrolat, 1833	2n = 20, 9 + Xyp	[34]
*Tetraonyx quadrimaculata* Fabricius, 1792	2n = 20, 9 + Xyp	[51]
**Subfamily Nemognathinae**		
*Zonitis tarasca* Dugès, 1888	2n = 20, 9 + Xyp	[34]

## Data Availability

Newly obtained sequences were deposited in GenBank, accession numbers MW711195 to MW711206.
